# Fermentation, Purification, and Tumor Inhibition of a Disulfide-Stabilized Diabody Against Fibroblast Growth Factor-2

**DOI:** 10.3389/fonc.2021.585457

**Published:** 2021-02-25

**Authors:** Simin Zhang, Jiahui Huang, Ligang Zhang, Jiangtao Gu, Qifang Song, Yaxiong Cai, Jiangchuan Zhong, Hui Zhong, Yanrui Deng, Wenhui Zhu, Jianfu Zhao, Ning Deng

**Affiliations:** ^1^ Guangdong Province Engineering Research Center for Antibody Drug and Immunoassay, Department of Biology, Jinan University, Guangzhou, China; ^2^ Biomedicine Translational Institute, Jinan University, Guangzhou, China; ^3^ Cancer Diagnosis and Therapy Research Center, Department of Oncology of the First Affiliated Hospital, Jinan University, Guangzhou, China

**Keywords:** fibroblast growth factor 2, angiogenesis, disulfide-stabilized diabody, melanoma cancer, glioma cancer

## Abstract

Angiogenesis is considered one of the hallmarks of cancer and plays a critical role in the development of tumor. Fibroblast growth factor 2 (FGF-2) is a member of the FGF family and participates in excessive cancer cell proliferation and tumor angiogenesis. Thus, targeting FGF-2 was considered to be a promising anti-tumor strategy. A disulfide-stabilized diabody (ds-Diabody) against FGF-2 was produced in *Pichia pastoris* (GS115) by fermentation and the anti-tumor activity was analyzed. The novel 10-L fed batch fermentation with newly designed media was established, and the maximum production of the ds-Diabody against FGF-2 reached 210.4 mg/L. The ds-Diabody against FGF-2 was purified by Ni^2+^ affinity chromatography and DEAE anion exchange chromatography. The recombinant ds-Diabody against FGF-2 could effectively inhibit proliferation, migration, and invasion of melanoma and glioma tumor cells stimulated by FGF-2. Furthermore, xenograft tumor model assays showed that the ds-Diabody against FGF-2 had potent antitumor activity in nude mice by inhibiting tumor growth and angiogenesis. The tumor growth inhibition rate of melanoma and glioma was about 70 and 45%, respectively. The tumor angiogenesis inhibition rate of melanoma and glioma was about 64 and 51%, respectively. The results revealed that the recombinant ds-Diabody against FGF-2 may be a promising anti-tumor drug for cancer therapy.

## Introduction

Malignant tumor has become the second leading cause of death worldwide, and there were about 9.6 million cancer-related deaths in the year before last (1). Angiogenesis is considered one of the hallmarks of cancer and plays a critical role in the development of tumor (2). These new blood vessels mainly transport nutrients, oxygen, and metabolic wastes for the growth and metastasis of tumors (3). Therefore, anti-angiogenesis is a very important strategy for cancer therapy designed to disrupt the vascular supply (4).

Fibroblast growth factor 2 (FGF-2) is a member of the FGF family that functions in regulating both normal and abnormal angiogenesis (5, 6). It is upregulated in inflammatory stimuli and in tumors (7–9). It has been considered that the active FGF-2 could mediate the formation of new blood vessels (10). FGF-2 integrates the extracellular domain of fibroblast growth factor receptor (FGFR) and heparan sulfate proteoglycans (HSPG) to induce the autophosphorylation of FGFR intracellular tyrosine kinase domain and lead to the activation of complex signal transduction pathways (11, 12). They participate in the development of tumors as oncogenes by inducing mitogenic and survival signals, promoting tumor cell invasion and metastasis, promoting epithelial–mesenchymal transition, promoting angiogenesis, and participating in tumor recurrence and drug resistance (13). Thus, targeting FGF-2 for inhibition of tumor growth and angiogenesis was considered to be a promising therapeutic strategy.

In the last decade, FGF/FGFR inhibitors were new focuses in the field of cancer therapy. The tyrosine kinase inhibitors (TKIs), such as TKI258, cediranib, and brivanib, can block receptor signaling through competitive inhibition of ATP binding with the cytoplasmic domain of EGFR, FGFR, and VEGFR, but they are less capable of achieving efficient inhibitions and also increasing side effects (14, 15). The other attractive option as a selective FGF/FGFR inhibitor is the monoclonal antibody (mAb). mAbs offer some significant advantages over TKIs, including specificity for targeting to a particular receptor. This circumvents certain toxicity issues and enhances the anti-tumor response (16). A disulfide-stabilized diabody (ds-Diabody) against FGF-2, a kind of small molecular monoclonal antibody, was composed of variable heavy chain (VH) and variable light chain (VL) with a short connecting linker (five amino acid residues) to induce formation of the scFv dimer, and its disulfide bonds were introduced to maintain stability and affinity. Small molecule antibodies get more and more attention for their good tissue penetration and low immunogenicity (17). They may have potential applications in tumor-targeted therapy.

In this study, we mainly reported the production of ds-Diabody against FGF-2 by high density fermentation in recombinant *Pichia pastoris* and its effects on tumor angiogenesis and tumor growth *in vitro* and *in vivo*.

## Materials and Methods

### Cell Culture

Human melanoma cancer cells (A375) (ATCC, Manassas, VA, USA) were cultured in RPMI-1640 medium (Gibco, Grand Island, NY, USA), and the human glioma cancer cells (U87) (ATCC, Manassas, VA, USA) were cultured in DMEM medium (Gibco) supplemented with 10% fetal bovine serum (FBS, Gibco) and 1% penicillin/streptomycin (Gibco) in an incubator at 37°C with 5% CO2.

### Animals

Female BALB/c nude mice (6–7 weeks old) were purchased from Beijing Sibeifu Biotechnology Co., Ltd. (China). Animals were acclimatized to the facilities for 10 days. The mice were housed in specific pathogen-free environment, and the treatment of animals at 22 ± 2°C and 50 ± 10% relative humidity with water and food (laboratory rodent chow, Shanghai, China) allowed *ad libitum*. The treatment of animals was approved by the Institutional Animals Care and Use Committee on animal research in Jinan University, Guangzhou, China.

### The Production of ds-Diabody Against FGF-2 by Fermentation

The recombinant *Pichia pastoris* GS115-pPICZαA-ds-Diabody constructed in our previous study (17) was inoculated into 300 ml of YPG medium (per liter contained tryptone, 20.0 g; Yeast extract, 10.0 g; glycerol, 10.0 g) and cultivated at 28°C with 250 rpm shaking for 36 h. The medium was transferred to 3 L of BSM medium (per liter contained histidine, 13.3 g; glycerol, 40.0 g; K_2_SO_4_, 18.0 g; MgSO_4_·7H_2_O, 14.9 g; KOH, 4.13 g; CaSO_4_, 0.9 g; H_3_PO_4_, 27.0 ml; H_2_SO_4_, 5.0 ml and 4.0 ml of PTM trace salts which was per liter consisted of CuSO_4_·5H_2_O, 6.0 g; KI, 0.09 g; MnSO_4_.H_2_O, 3.0 g; H_3_BO_3_, 0.02; MoNa_2_O_4_·2H_2_O, 0.2 g; CoCl_2_, 0.5 g; ZnCl_2_, 20.0 g; FeSO_4_·7H_2_O, 65.0 g and biotin, 0.2 g) in a 10-L standard mechanical agitated fermenter bioreactor (T&J Bio-engineering Co., Ltd, Shanghai, China). At the stage of fed-batch cultivation (phase I), the culture was maintained at 28°C, pH 5.0 and 20% dissolved oxygen (DO). After 18–24 h, glycerol was exhausted, and the DO increased to 100% rapidly. The fed-batch phase (phase II) was initiated by feeding limited glycerol to allow further cell growth. 50% (v/v) glycerol supplemented with PTM trace salts (12 ml/L) was added at a rate of 12 ml/h/L. The methanol fed-batch (strategy I) or the sorbitol/methanol fed-batch (strategy II) was taken in phase III after the cell wet weight reached 180–220 g/L. For strategy I, methanol was added with PTM trace salts (12 ml/L) at the speed of 1 ml/h/L, and the feeding speed of methanol increased by a ratio of 20% per hour until it reached 6 ml/h/L. For strategy II, the sorbitol was co-fed with methanol at a fixed feeding ratio of 1:4 (g/g). In phase III, the condition of fermentation was adjusted to 28°C, pH 6.0 and the DO ≥20%. The cell wet weight was measured, and the supernatant was collected by centrifuging the culture at 12,000 g for 15 min at 4°C. The expression of ds-Diabody was analyzed by reduced SDS-PAGE.

### Purification of ds-Diabody Against FGF-2

The supernatant was precipitated with ammonium sulfate and dialyzed against a binding buffer. It was purified by Ni^2+^-affinity chromatography due to His-tag at C-terminal of recombinant protein and by DEAE weak-anion exchange chromatography for higher purity according to the manufacture protocol. The eluted fractions were dialyzed with phosphate buffered saline (PBS) using 10-kDa cut-off membrane ultrafiltration devices (GE healthcare) and stored at −20°C for further analysis.

### SDS-PAGE Electrophoresis Analysis

The molecular mass of ds-Diabody was determined by reduced sodium dodecyl sulfate polyacrylamide gel electrophoresis (SDS-PAGE). Each sample was mixed with 5× loading buffer (Sangon, Shanghai, China) and heated for 10 min at 100°C. Samples were then separated under reducing condition on 12% (m/v) tris-glycine gels (Sangon) using a Mini-gel system (Bio-Rad). The proteins in the gel were stained with Coomassie (Sangon) and analyzed using the Automatic Analysis System of Electrophoresis Gel Imaging (Tanon Science Technology Co. Ltd, Shanghai, China).

## Identification of ds-Diabody Against FGF-2 by Western Blot

After SDS-PAGE, the proteins were transferred onto polyvinylidenedifluoride (PEDV) membrane (Millipore, Bedford, USA). The membrane was block with 5% (m/v) non-fat milk dissolved in phosphate buffered saline supplemented with 0.05% (v/v) Tween-20 (PBS-T) at 37°C for 2 h and probed with anti-His tag antibody (1:1,000 dilution) (362601, Biolegend, San Diego, CA) at 4°C overnight, followed by incubation with the horseradish peroxidase (HRP) conjugated goat anti-mouse IgG (1:8,000 dilution) (405306, Biolegend) at 37°C for 30 min. The membrane was washed three times for 3 min with PBS-T between incubation steps. Immunoreactive bands were visualized by Immobilon Western Chemiluminescent HRP Substrate (Millipore, Bedford, USA) according to protocol and were performed with Automatic Gel Image Analysis System (Tanon Science Technology Co. Ltd).

### Antigen Binding Activity of ds-Diabody Against FGF-2 by ELISA

The 96-well plates were coated with FGF-2 (50 ng/well) (#P09038, R&D, Minneapolis, MN, USA) at 4°C overnight, and the BSA (50 ng/well) (A3311, Sigma) coated wells were control group for non-specific protein binding. The next day, the plates were washed and blocked with 5% non-fat milk at 37°C for 2 h. The purified ds-Diabody was quantified by BCA Protein Assay Kit (Thermo Scientific, Rockford, IL, USA) according to the protocol and was serially diluted (15.63, 7.81, 3.91, 1.95, 0.98, 0.49, 0.24, 0.12, 0.06, 0.03, and 0.02 μg/ml) with PBS buffer and incubated at 37°C for 1.5 h. The anti-c-Myc antibody (1:2,000 dilution) (sc-40, Santa Cruz, Cambridge, UK) was incubated at 37°C for 1.5 h binding to c-Myc tag of ds-Diabody. The HRP-conjugated goat anti-mouse IgG (1:4,000 dilution) (405306, Biolegend, San Diego, CA) was added and incubated at 37°C for 30 min. The plates were washed three times for 3 min with PBS-T between incubation steps. The reaction wells were developed with TMB (3,3′,5,5′-tetramethylbenzidine) (Sigma, St. Louis, MO, USA), and the reaction was stopped with sulfuric acid. The absorbance values of each well were measured at 450 nm using the ELISA reader (BioTek, Highland Park, Winooski, VT, USA).

### Cell Proliferation Assays

The human melanoma cancer cells (A375) (2,000 cells/well) and the human glioma cancer cells (U87) (1,000 cells/well) were seeded in 96-well plates and incubated overnight at 37°C in a 5% CO_2_ incubator. The cells were then serum-starved cultured in the medium with 0.5% FBS for 12 h and treated with serially diluted ds-Diabodies (6.25–100 μg/ml) with 15 ng/ml FGF-2 for 48 h. The full-length human IgG against FGF-2 (6.25–100 μg/ml) was the positive control, and control IgG (6.25–100 μg/ml) was negative control. The proliferation of tumor cells was assayed using Cell Counting Kit-8 (CCK-8) (Dojindo Laboratories, Kumamoto, Japan) according to the manufacturer’s protocol. The proliferation inhibition rate was calculated according to the OD_450_ values measured in an ELISA reader (BioTek).

### Migration Inhibition of ds-Diabody on Human Melanoma and Glioma Cells

The effects of the ds-Diabody against FGF-2 on migration of A375 cells and U87 cells were evaluated by wound healing assays. A375 cells (2.5 × 10^5^ cells/well) and U87 cells (2.0 × 10^5^ cells/well) were seeded in 6-well plates and incubated at 37°C for 12–24 h in a 5% CO_2_ incubator. When cells grew to confluence, a cell-free area was introduced using a pipette tip. After that, cells were washed with PBS, cultured in the medium with 0.5% FBS and treated with ds-Diabody (100 μg/mL) with 15 ng/mL FGF-2. The full-length human IgG against FGF-2 (100 μg/ml) was positive control, control IgG (100 μg/ml) was negative control, and PBS was vehicle control, respectively. Cells were captured at 0, 12, and 24 h and the migration rate was calculated.

### Invasion Assays

The effects of the ds-Diabody against FGF-2 on invasion of A375 cells and U87 cells were evaluated by invasion assays. The transwell chamber with 8 μm filter (BD Biosciences, San Jose, CA, USA) was coated with 100 μl Matrigel matrix (BD Biosciences) which was diluted (1:30) with serum-free medium. After serum-starved for 12 h, A375 cells (2.0 × 10^4^ cells/well) and the U87 cells (1.0 × 10^4^ cells/well) were transferred to the transwell chambers and cultured in serum-free medium with 15 ng/ml FGF-2 and treated with ds-Diabody (100 μg/ml) for 16 h. 600 μl medium with 10% FBS was added in the lower chambers as a chemoattractant. After the treatment, cells on the lower side of the filter were fixed with 70% ethanol, stained by 0.1% crystal violet (Meryer, Shanghai, China) and imaged with a computerized imaging system. Full-length human IgG against FGF-2 (100 μg/ml), control IgG (100 μg/ml), and PBS were positive control, negative control, and vehicle control, respectively.

### Western Blot Assays of Phosphorylation of Akt and MAPK

A375 cells (2.0 × 10^5^ cells/well) and U87 cells (1.0 × 10^5^ cells/well) were seeded in 6-well plates and incubated at 37°C overnight in a 5% CO_2_ incubator. Cells were serum-starved cultured in the medium with 0.5% FBS for 12 h. The medium was exchanged containing 15 ng/ml FGF-2, 0.5% FBS, and cells were treated with serially diluted ds-Diabody (0, 50, 100, and 200 μg/ml) at 37°C for 30 min. Cells were then washed with PBS and lysed with RIPA lysis buffer (Beyotime Biotechnology, Suzhou, China) at 4°C for 10 min. The lysates were centrifuged at 12,000 g for 10 min at 4°C. The total proteins in supernatant were quantified by using Pierce BCA Protein Assay Kit (Thermo Scientific). Proteins were separated by reduced SDS-PAGE and transferred to PVDF membrane (Millipore). The membrane was blocked with 5% non-fat milk at 37°C for 2 h and incubated with rabbit anti-t/p-MAPK (Cell Signaling Technology, Danvers, MA, USA) and rabbit anti-t/p-Akt (Cell Signaling Technology) at 4°C overnight. The membrane was then incubated with the HRP-conjugated goat anti-rabbit IgG (Cell Signaling Technology) for 30 min at 37°C. The blots were detected with the Immobilon Western Chemiluminescent HRP Substrate (Millipore) according to manufacturer’s protocol. The rabbit anti-GAPDH antibody (Cell Signaling Technology) was used as reference control.

### Tumor Xenograft Models

For melanoma and glioma tumor xenograft experiments, BALB/c-nu mice (n = 7) were randomly assigned into three treatment groups (seven mice per group). A375 cells (2.0 × 10^6^ cells in 100 μl) and U87 cells (5.0 × 10^6^ cells in 100 μl) were s.c. injected into the right shoulder of female BALB/c nude mice. When tumors were palpable, mice were i.v. injected with the ds-Diabody (5 mg/kg in 100 μl PBS) six times at 3 days intervals. Avastin antibody (2 mg/kg in 100 μl PBS) and PBS (100 μl) were positive control and vehicle control, respectively. Body weights and tumor sizes were measured every 3 days. Tumor volumes (mm^3^) were calculated according to the formula V = 0.52ab^2^ (a = length of tumor; b = width of tumor). At the end of 15 days, nude mice were euthanized, and tumors were stripped for weighing to calculate tumor growth inhibition rate and for immunohistochemistry assays. Tumor growth inhibition rates were calculated as (1 − the average tumor weight of treated groups/the average tumor weight of PBS group) × 100%.

### Immunohistochemistry Assays for CD31

The immunohistochemistry assays for CD31 were performed to determine the intratumoral microvessel density. CD31 was detected in immersion fixed paraffin sections of tumor tissues using anti-CD31 rabbit polyclonal antibodies (ab28364; Abcam, Cambridge, UK) at 10 µg/ml overnight at 4°C followed by incubation with goat anti-rabbit IgG H&L HRP Polymer Antibody (ab205718; Abcam, Cambridge, UK). Tissues were stained using DAB (brown) and counterstained with hematoxylin (blue).

### Statistical Analysis

Data were expressed as the mean and standard deviation (SD). P <0.05 was considered statistically significant. Assays were performed at least three times independently. Statistical comparisons between two groups were analyzed by one-way ANOVA with the least significant difference test (LSD test).

## Results

### The Production of ds-Diabody Against FGF-2 by Fermentation

**Figure 1:**
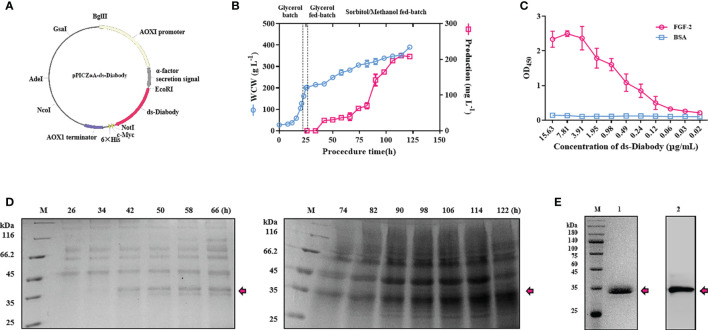
The fermentation, purification and binding activity studies of ds-Diabody against FGF-2 in Pichia pastoris. **(A)** Schematic illustration of the recombinant expression plasmid of pPICZαA-ds-Diabody. **(B)** The curve of cell wet weight and the production of ds-Diabody against FGF-2 in *Pichia pastoris* fermentation for strategy II. **(C)** The antigen combined activity of ds-Diabody against FGF-2 by ELISA. **(D)** The SDS-PAGE assays of ds-Diabody against FGF-2 in fermentation for strategy II. **(E)** The purification of ds-Diabody. Lane M, marker; Lane 1, the SDS-PAGE assay of the purified ds-Diabody in reducing condition; Lane 2: the Western blot assays of the purified ds-Diabody.

The recombinant *Pichia pastoris* GS115-pPICZαA-ds-Diabody was constructed in our previous study (**Figure 1A**) (17). The maximum production ds-Diabody was 47.4 mg/L and wet cell weight (WCW) was 393.1 g/L in methanol fed-batch (strategy I) (**Figure S1**). *the Pichia pastoris* expression system is impressive and easy to handle, and the process optimizations are required to achieve maximum production of the target proteins especially in induction phase. Among the co-feeding strategies, co-carbon source (sorbitol) feeding strategy is as important as methanol feeding strategy (18). Thus, in order to further improve the production of the target protein, sorbitol/methanol fed-batch (strategy II) was used in our study. Surprisingly, the maximum ds-Diabody concentration reached 210.4 mg/L and WCW was 390.0 g/L in sorbitol/methanol fed-batch phase (**Figures 1B**). The production of ds-Diabody was increased by 4.4 times when mixed feeding of sorbitol and methanol. The ds-Diabody expressed successfully and was observed a band of about 35 kDa using reduced SDS-PAGE (**Figure 1D**).

### Purification of ds-Diabody Against FGF-2

The supernatant was precipitated with ammonium sulfate and dialyzed against a binding buffer. Supernatant was then purified through Ni^2+^-affinity chromatography due to His-tag at C-terminal of recombinant protein and through anion-exchange chromatography for higher purity. The purified recombinant protein was observed as a single band of about 35 kDa using reduced SDS-PAGE and western blot analysis with >95% purity (**Figure 1E**).

### Antigen Binding Activity of the ds-Diabody Against FGF-2 Using Indirect ELISA Assays

To further determine the binding activity and specificity of ds-Diabody, the indirect ELISA assays were performed. When the concentration of the ds-Diabody was 0.49 μg/ml, the value of OD_450_ in FGF-2 group was 1.08, while 0.12 in BAS group. The results demonstrated that ds-Diabody bound specifically to FGF-2 and had a high binding ability (**Figure 1C**).

### Proliferation Inhibition of ds-Diabody on Human Melanoma and Glioma Cells

**Figure 2:**
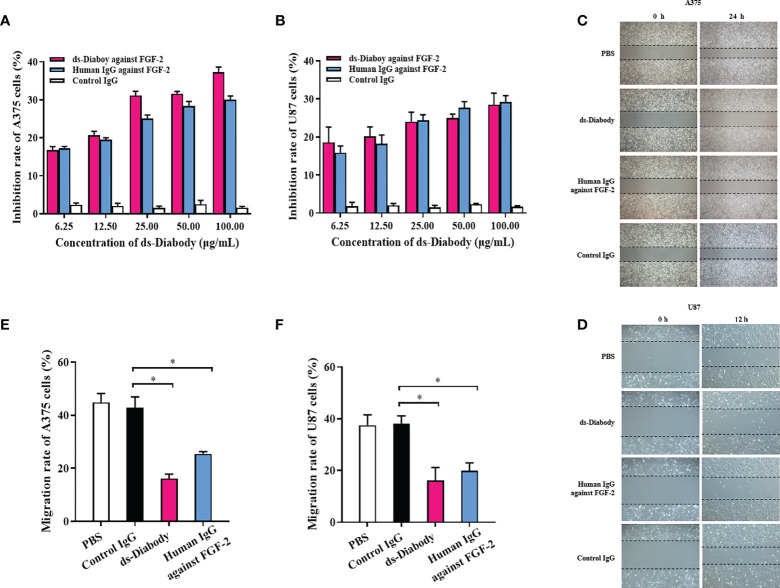
The tumor proliferation and migration inhibition of ds-Diabody against FGF-2. **(A)** The proliferation inhibition of ds-Diabody on A375 cells. **(B)** The proliferation inhibition of ds-Diabody on U87 cells. **(C)** The migration of A375 cells in different groups. **(D)** The migration of U87 cells in different groups. **(E)** The quantitative analysis of the migration rate of A375 cells in different groups. **(F)** The quantitative analysis of the migration rate of U87 cells in different groups. The results presented as the mean ± SD in triplicate. *p < 0.05 *versus* Irrelevant Group.

A375 cells and U87 cells were shown to express FGF-2 at high levels (19–21). Additionally, FGF-2 strongly stimulates the proliferation and migration of tumor cells (5). To investigate bioactivity of the ds-Diabody to inhibit FGF-2-induced proliferation of *in vitro*, CCK-8 assays were performed. The results showed that ds-Diabody displayed dose-dependent inhibitory activity on A375 cells and U87 cells. When the concentration of the ds-Diabody was 6.25, 12.5, 25.0, 50.0 and 100.0 μg/ml, the A375 cells’ proliferation inhibition rate was about 16, 21, 30, 32, and 37%, respectively (**Figure 2A**); the U87 cells’ proliferation inhibition rate was about 19, 20, 24, 25, and 29%, respectively (**Figure 2B**), which showed similar efficacy to full-length human IgG (positive control). Control IgG showed no inhibitory effect.

### Migration Inhibition of ds-Diabody on Tumor Cells

Wound healing assays were used to reveal the effects of ds-Diabody on migration of A375 cells and U87 cells. A375 cells (2.5 × 10^5^ cells/well) and U87 cells (2.0 × 10^5^ cells/well) were seeded in 6-well plates and incubated at 37°C in a 5% CO2 incubator. When cells grew to confluence, a cell-free area was introduced using a pipette tip, and cells were cultured in the medium with 0.5% FBS and treated with ds-Diabody (100 μg/ml) with 15 ng/ml FGF-2. The migration rate of A375 cells for 24 h in the group of the ds-Diabody, the full-length human IgG against FGF-2, the irrelevant IgG and PBS were 16, 25, 43, and 45%, respectively (**Figures 2C, E**). The migration rate of U87 cells for 12 h in groups of the ds-Diabody against FGF-2, the full-length human IgG against FGF-2, the control IgG and PBS were 16, 20, 38, and 38%, respectively (**Figures 2D, F**). It was shown that the migration of A375 and U87 cells were significantly inhibited in ds-Diabody-treated group compared to control.

### Invasion Inhibition of ds-Diabody on Tumor Cells

**Figure 3:**
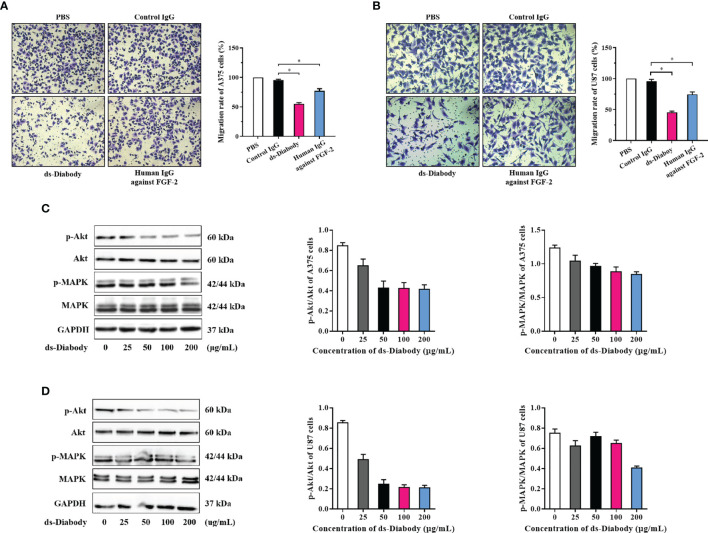
The invasion inhibition and phosphorylation assays of Akt and MAPK in tumor cells treated with ds-Diabody against FGF-2. **(A)** The invasion and the quantitative analysis of A375 cells in different groups. **(B)** The invasion and the quantitative analysis of U87 cells in different groups. **(C)** Western blot assays of Akt and MAPK phosphorylation in A375 cells treated by ds-Diabody against FGF-2. **(D)** Western blot assays of Akt and MAPK phosphorylation of U87 cells treated by ds-Diabody against FGF-2. The results presented as the mean ± SD in triplicate. *p < 0.05 *versus* Irrelevant Group.

The invasion assays were used to reveal the effects of the ds-Diabody on invasion of A375 cells and U87 cells. After serum-starved for 12 h, A375 cells (2.0 × 10^4^ cells/well) and the U87 cells (1.0 × 10^4^ cells/well) were transferred to the transwell chambers. Cells cultured in upper chamber with serum-free medium and 15 ng/ml FGF-2 were chemo-attracted to the lower chamber containing complete medium. The invasion rate of A375 cells in the group of the ds-Diabody, the full-length human IgG against FGF-2, the control IgG and PBS were 55, 77, 96, and 100%, respectively (**Figure 3A**). The invasion rate of U87 cells in the group of the ds-Diabody, the full-length human IgG against FGF-2, the control IgG and PBS were 46, 75, 96, and 100%, respectively (**Figure 3B**). It was shown that the ds-Diabody could significantly inhibit the invasion of A375 cells and U87 cells.

### Western Blot Assay of Phosphorylation of Akt and MAPK

FGF-2 could promote tumor proliferation, migration, and angiogenesis as a key angiogenic factor (5). To further confirm whether ds-Diabody was capable of inhibiting FGF-2 signaling, a phosphorylation assay of Akt and MAPK was performed. The starved A375 cells and U87 cells were treated with different concentrations of ds-Diabody together with FGF-2. The cells were then lysed, and proteins in lysates were separated with reduced SDS-PAGE and detected by western-blot. The results showed that the ds-Diabody could effectively block the phosphorylation activation of Akt and MAPK in a dose-dependent manner (**Figures 3C, D**), which suggesting an effective inhibitory function of ds-Diabody against FGF-2-stimulated proliferation, migration and angiogenesis in A375 cells and U87 cells.

### The ds-Diabody Against FGF-2 Suppressed Tumors Growth and Angiogenesis in Xenograft Models

**Figure 4:**
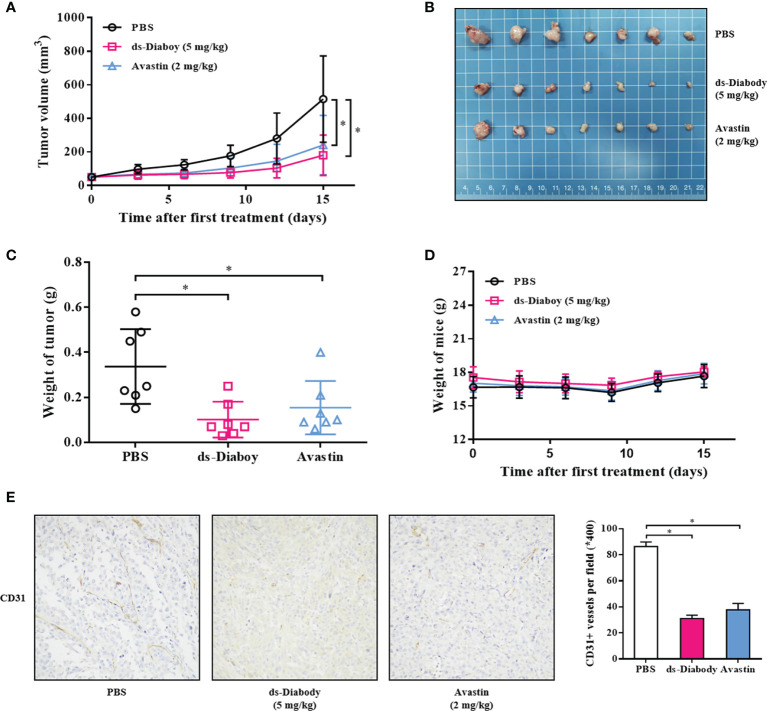
The tumor inhibition of melanoma by ds-Diabody against FGF-2 in mice. The melanoma (A375) cells (2.0 × 10^6^ cells in 100 μl medium) were subcutaneously injected on the shoulder of BALB/c nude mice (n = 7). When the tumors were palpable, the ds-Diabody against FGF-2 were i.v. injected (5 mg/kg in 100 μl PBS) six times at 3 days intervals. **(A)** The tumor growth curve in nude mice in different groups. **(B)** The stripped tumors from nude mice in different groups. **(C)** The scatter diagram of tumor weights in different groups. **(D)** The weight curves of nude mice in different groups. **(E)** Immunohistochemistry analysis of microvessels of tumor tissues. The results presented as the mean ± SD in triplicate. *p < 0.05 *versus* PBS Group.

**Figure 5:**
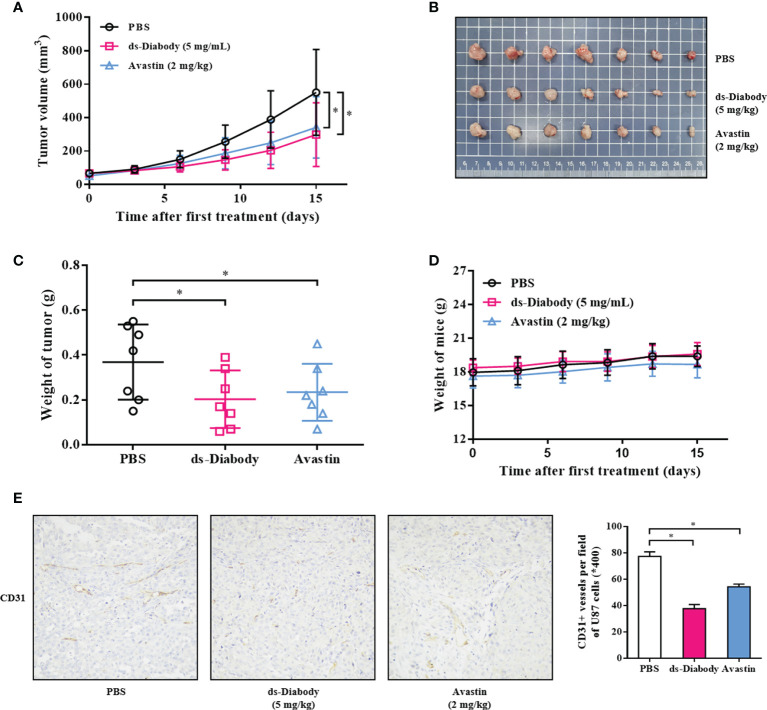
The tumor inhibition of glioma by ds-Diabody against FGF-2 in mice. The glioma (U87) cells (5.0 ×10^6^ cells in 100 μl medium) were subcutaneously injected on the shoulder of BALB/c nude mice (n = 7). When the tumors were palpable, the ds-Diabody against FGF-2 were i.v. injected (5 mg/kg in 100 μL PBS) six times at 3 days intervals. **(A)** The tumor growth curve in nude mice in different groups. **(B)** The stripped tumors from nude mice in different groups. **(C)** The scatter diagram of tumor weights in different groups. **(D)** The weight curves of nude mice in different groups. **(E)** Immunohistochemistry analysis of microvessels of tumor tissues. The results presented as the mean ± SD in triplicate. *p < 0.05 *versus* PBS Group.

The antitumor activity of ds-Diabody was evaluated in xenograft mouse models. The model was established *via* subcutaneously injection of A375 cells (2.0 × 10^6^ cells) or U87 cells (5.0 × 10^6^ cells) on the shoulder of female BALB/c nude mice. The result showed that the tumor volumes and tumor weights of the ds-Diabody-treated group were significantly lower compared to the control group. The growth inhibition rate of A375 tumor in the ds-Diabody group was about 70% and in the Avastin group was about 54% (**Figures 4A–C**). The growth inhibition rate of U87 tumor in the ds-Diabody group was about 45% and in the Avastin group was about 36% (**Figures 5A–C**). The weights of BABL/c-nu mice treated with ds-Diabody were relatively stable (**Figures 4D**, **5D**). Moreover, immunohistochemical analysis showed that ds-Diabody could reduce the expression of CD31 in A375 (**Figure 4E**) and U87 (**Figure 5E**) tumor tissues. The angiogenesis inhibition rate of A375 tumor and U87 tumor treated by ds-Diabody was about 64 and 51%, respectively, suggesting that ds-Diabody could suppress tumor growth by inhibiting tumor angiogenesis.

## Discussion

Currently, several expression systems are used to produce recombinant proteins such as bacteria, yeasts and mammals (22). Compare to bacteria expression systems, yeast expression systems like *Pichia pastoris* are preferred tools for high expression of properly folded secretory proteins and limited production of endogenous secretory proteins (23, 24). Moreover, they are easy to handle, less costly, and well suited for fermentation processes (25). Mammalian cell lines such as CHO cells can realize proper protein folding, posttranslational modifications, and glycosylation of recombinant proteins, but the expensiveness, long term culture and potential contamination with some viruses limited their wide use (26). Although *the Pichia pastoris* expression system is impressive and easy to use with well-defined process protocol, some degree of process optimization is required to achieve maximum production of the target protein.

The optimization in induction phase using substrate mixtures is crucial for improving the productivity of heterologous proteins with *Pichia pastoris*. Among the co-feeding strategies, co-carbon source (sorbitol) feeding strategy is as important as methanol feeding strategy. Sorbitol does not induce or repress AOX promoters; hence using sorbitol instead of glycerol co-fed with methanol could increase productivity and reduce induction time (18). In addition, the formation of toxic formaldehyde was reduced, and the cellular viability was enhanced by sorbitol/methanol co-feeding (27). Several studies revealed that methanol/sorbitol co-feeding increased the expression of the recombinant proteins (28–31). Consistent with this, using sorbitol in mixed substrate methods in our study, the induction time was reduced, and the production of ds-Diabody against FGF-2 was increased by 4.4 times.

FGF-2/FGFR system contributes to cancer progression by autocrine/paracrine stimulation of tumor cell proliferation and angiogenesis (32, 33). Elevated concentrations of FGF-2 and increased tumor microvascular density are potential markers of worse overall prognosis and abbreviated progression-free survival (19). FGF-2 was overexpressed in several tumors including melanoma and glioma (19–21). In this study, our data showed that ds-Diabody against FGF-2 could effectively inhibit melanoma and glioma *in vivo*. Li et al. (2018) suggested that LncRNA MEG3 can produce an almost 50% decrease in A375 tumor growth through blocking Wnt signaling pathway (34). Zhou et al. found that GHRH antagonists produced an almost 60% decrease in U87 tumor growth by multiple mechanisms including decreasing the release of FGF (35). Our data showed that the growth inhibition rate of A375 tumor and U87 tumor was about 70 and 45%, respectively, and the angiogenesis inhibition rate of A375 tumor and U87 tumor was about 64 and 51%, respectively. The inhibition effects of tumor growth and angiogenesis showed more effective compared to the Avastin group, which suggested that ds-Diabody was a potential anti-tumor drug for tumor therapy.

To identify the inhibition mechanism of ds-Diabody on the growth of A375 and U87 cells, the potential signal transduction pathways were detected. It was shown that the ds-Diabody against FGF-2 could effectively inhibit the proliferation, migration, and invasion of the A375 and U87 cells and block the phosphorylation activation of Akt and MAPK in a dose-dependent manner. MAPK/ERK and PI3K/Akt are important signaling pathways in a variety of cellular processes, including cell proliferation and migration in tumor cells (36). Currently, several available inhibitors against the components of MAPK/ERK pathway have led to unprecedented clinical benefits in combating metastatic melanoma and glioma (37). Previous study showed that drugs targeting the MAPK/ERK pathway have profoundly changed the landscape of melanoma therapy (38). Similarly, several studies reported that MAPK/ERK signaling pathway involves in the migration and invasion of glioma cells (37). In our study, we found that MAPK/ERK and PI3K/AKt signals were significantly inhibited by the ds-Diabody. Therefore, our study discovered a possible upstream event through which ds-Diabody exerted its anti-melanoma and glioma activity, although the precise mechanism needs further investigation.

Pathological angiogenesis plays a critical role in cancer and the anti-angiogenesis treatment has been also considered as a systemic strategy for cancer therapy (39). FGF-2 is an important tumor angiogenesis factor, and targeting FGF-2 can inhibit tumor angiogenesis and tumor growth. In our study, it was shown that the expression of CD31, a marker of vascularity, was decreased obviously in tumor tissues treated by ds-Diabody against FGF-2. The ds-Diabody against FGF-2 might possess significant effect on tumor therapy, compared to Avastin, and could be considered as a potential candidate for cancer therapy.

To summarize, the present study reported a fully human ds-Diabody against FGF-2 expressed in *Pichia pastoris*, which possessed high antigen binding ability and anti-tumor activity *in vitro* and *in vivo* through inhibiting angiogenesis. Further studies will be necessary to study the ds-Diabody mechanisms and compare the efficacy with Avastin in other xenograft models. Based on its high efficiency and low toxicity, ds-Diabody against FGF-2 may provide an effective strategy for cancer therapy.

## Conclusion

In conclusion, co-feeding of sorbital and methanol during the induction phase of *Pichia pastoris* fermentation was more favorable than feeding of methanol and resulted in higher production of ds-Diabody against FGF-2. The ds-Diabody against FGF-2 inhibited the proliferation, migration, and invasion of A375 cells and U87 cells by decreasing the MAPK/Akt signal pathways. Additionally, in xenograft models, ds-Diabody against FGF-2 effectively suppressed melanoma and glioma tumo growth and angiogenesis. The ds-Diabody targeting FGF-2 may provide an effective strategy for cancer therapy.

## Data Availability Statement

The raw data supporting the conclusions of this article will be made available by the authors, without undue reservation.

## Ethics Statement

The animal study was reviewed and approved by Institutional Animals Care and Use Committee on animal research in Jinan University, Guangzhou, China.

## Author Contributions

SZ, JH, and LZ designed and performed the experiments and drafted the manuscript. JG participated in the experiments. QS, YC, JCZ, HZ, YD, and WZ were involved in resource collection. ND and JFZ supervised this study. All authors contributed to the article and approved the submitted version.

## Funding

This research was supported by grants from The Natural Science Foundation of China (81972705), Science and Technology Planning Project of Guangdong Province (2015B020211009, 2016A010105008) and Science and Technology Planning Project of Guangzhou City (201604020099).

## Conflict of Interest

The authors declare that the research was conducted in the absence of any commercial or financial relationships that could be construed as a potential conflict of interest.
