# Supplementary material for: Fermentation, Purification, and Tumor Inhibition of a Disulfide-Stabilized Diabody Against Fibroblast Growth Factor-2
Source: Front Oncol. 2021 Feb 25;11:585457. doi: 10.3389/fonc.2021.585457 (PMC7947002; doi:10.3389/fonc.2021.585457)
Supplement: Supplementary file 1 [file DataSheet_1.doc]

**Fermentation, purification and tumor inhibition of a disulfide-stabilized diabody against fibroblast growth factor-2**

**Simin Zhang^1, #^, Jiahui Huang^1^****^, #^, Ligang Zhang****^1,#^, Jiangtao Gu^1^, Qifang Song^1^, Yaxiong Cai^1^, Jiangchuan Zhong^1^, Hui zhong^2^, Yanrui Deng^1^, Wenhui Zhu^1^, Jianfu Zhao^3 *^, Ning Deng^1 *^**

^1^Guangdong Province Engineering Research Center for Antibody Drug and Immunoassay, Department of Biology, Jinan University, Guangzhou, China

^2^ Biomedicine Translational Institute in Jinan University

^3^ The Oncology Department in the First Affiliated Hospital of Jinan University

***Correspondence：**

Ning Deng, e-mail: [tdengn@jnu.edu.cn](mailto:tdengn@jnu.edu.cn).

Jianfu Zhao: [zhaojianfu@jnu.edu.cn](mailto:zhaojianfu@jnu.edu.cn)

^#^The authors contributed equally to this work

**Supplementary figures and figure legends**


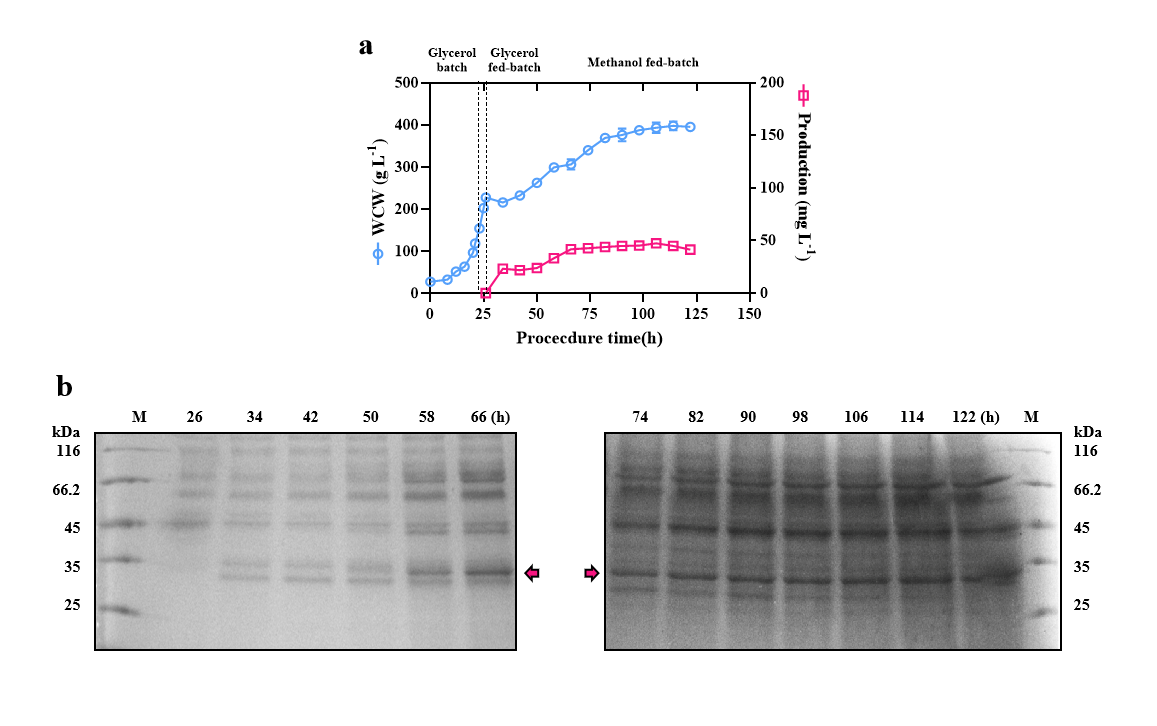


***Supplementary Figure 1. The fermentation of ds-Diabody against FGF-2 in Pichia pastoris***

**a**. The curves of cell wet weight and the production of ds-Diabody against FGF-2 in *Pichia pastoris* fermentation for strategy I. **c**. The SDS-PAGE assays of ds-Diabody against FGF-2 in fermentation for strategy I.
